# Using the *Galleria mellonella* burn wound and infection model to identify and characterize potential wound probiotics

**DOI:** 10.1099/mic.0.001350

**Published:** 2023-06-22

**Authors:** Evgenia Maslova, Shanga Osman, Ronan R. McCarthy

**Affiliations:** ^1^​ Division of Biosciences, Department of Life Sciences, Centre of Inflammation Research and Translational Medicine, College of Health, Medicine and Life Sciences, College of Health and Life Sciences, Brunel University London, Uxbridge, UK

**Keywords:** burn wound, burn infection, burn wound model, *Galleria mellonella*, *Pseudomonas aeruginosa*, probiotic, *Lactobacillus*

## Abstract

Burn wound infection is the leading cause of mortality among burn wound patients. One of the most commonly isolated bacterial burn wound pathogens is *Pseudomonas aeruginosa *, a notorious nosocomial multidrug-resistant pathogen. As a consequence of its recalcitrance to frontline antibiotic therapy, there is an urgent need to develop alternative treatment avenues to tackle this pathogen. One potential alternative infection prevention measure is to seed the wound bed with probiotic bacteria. Several species of *Lactobacillus,* a common commensal bacterium, have been previously reported to display growth inhibition activity against wound pathogens. Various species of this genus have also been shown to augment the wound healing process, which makes it a promising potential therapeutic agent. Due to the complexity of the burn wound trauma and burn wound infection, an *in vivo* model is required for the development of novel therapeutics. There are multiple *in vivo* models that are currently available, the most common among them being the murine model. However, mammalian burn wound infection models are logistically challenging, do not lend themselves to screening approaches and come with significant concerns around ethics and animal welfare. Recently, an invertebrate burn wound and infection model using *G. mellonella* has been established. This model addresses several of the challenges of more advanced animal models, such as affordability, maintenance and reduced ethical concerns. This study validates the capacity of this model to screen for potential wound probiotics by demonstrating that a variety of *Lactobacillus* spp*.* can limit *P. aeruginosa* burn wound infection and improve survival.

## Introduction

Burn wounds can cause significant damage to the integrity of the skin, exposing the affected individual to potential pathogens while also increasing local fluid loss [1]. As a consequence, more than 250 000 deaths worldwide are attributed to fire-induced burns alone, with a vast majority of those taking place in developing countries, where mortality among patients with 40 % of total body surface area (TBSA) reaches 100% [2]. Burn wound infection is the most prevalent complication of burn wound care and is the leading cause of mortality among burn wound patients [3]. In addition, infection can delay healing and lead to autograft failure ultimately resulting in longer treatment courses and hospital stays. This puts a significant burden on healthcare systems, which makes burn research a priority. In 2012–2013 UK’s National Health Service (NHS) was estimated to have managed nearly 90 000 burn injuries resulting in a £90 million cost [4]. One of the most frequently isolated bacteria in burn wounds is *Pseudomonas aeruginosa *, which is a Gram-negative opportunistic biofilm-forming pathogen [5]. *P. aeruginosa* is a highly virulent pathogen associated with high mortality rates and frequent outbreaks in burn ICUs [6–8]. The recalcitrance of this pathogen to front-line antibiotic therapy and its potential for causing localized outbreaks in burn treatment centres means novel therapeutic strategies are urgently needed to tackle this pathogen [9].

Several commensal probiotic bacteria, which are present on human skin, have been studied in the context of the burn wound healing and infection treatment. One of the most well-known and established probiotic bacteria is *Lactobacillus* (some species have been renamed to *Lactiplantibacillus* spp. and *Limosilactobacillus* spp.). *Lactiplantibacillus plantarum* (previously known as *Lactobacillus plantarum *) has been shown to decrease the bacterial load in infected burn wounds and improve wound healing in humans [10]. It has also been linked to reducing scar formation in the rabbit burn wound and infection model. *L. plantarum* probiotic therapy reduced the ability of *P. aeruginosa* to establish and maintain colonization of the wound and improved the skin restoration at the wound site [11]. *Lactobacillus* spp. populated alginate gels have also been used to prevent burn wound infection in rats [12]. Improved survival after *Lactobacillus acidophilus* and *Limosilactobacillus reuteri* (previously known as *Lactobacillus reuteri *) treatment has been observed in *Acinetobacter baumannii* infections in the mouse burn wound model [13]. *L. reuteri* has also exhibited protective properties towards epidermal keratinocytes in a *Staphylococcus aureus ex vivo* burn wound infection [14]. *Limosilactobacillus fermentum* (previously known as *Lactobacillus fermentum *) has also been shown to reduce the bioburden of *P. aeruginosa* in the murine burn wound infection model [15]. Overall, *Lactobacillus* spp. is one of the most promising candidates for probiotic-based therapies for burn wound infection. However, the further pre-clinical development of potential probiotic burn wound prophylaxis or treatment is being stymied by the complexities of conducting *in vivo* burn wound research. This is also limiting further insights into the underlying molecular mechanisms of how *Lactobacillus* spp. can prevent or limit burn wound infection.

**Fig. 1.**
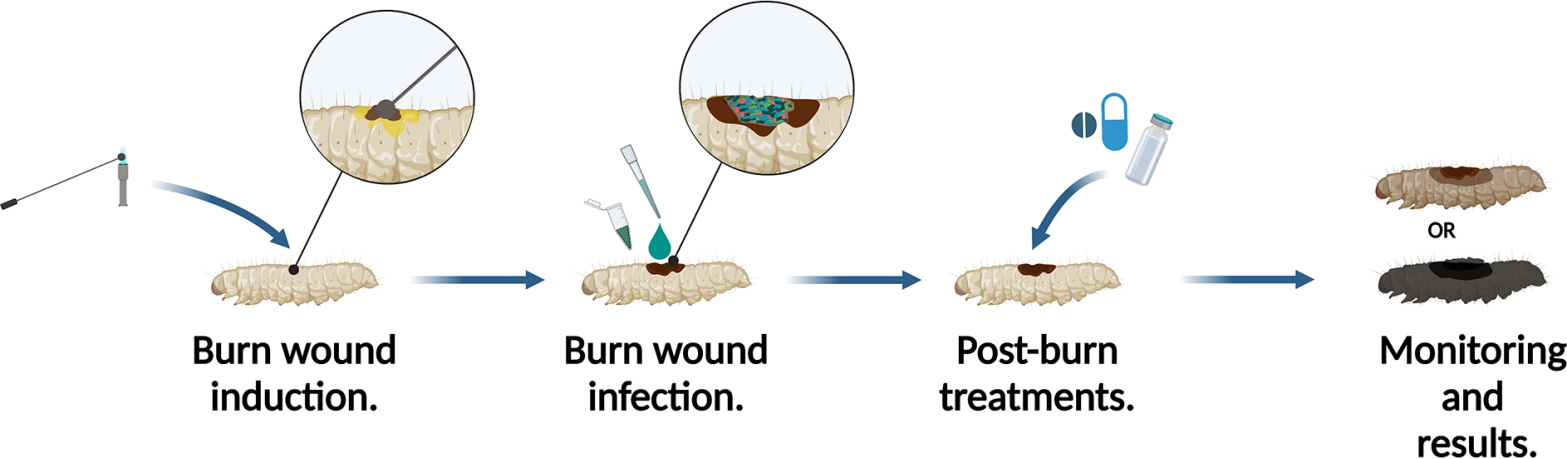
Schematic representation of *G. mellonella* burn wound and infection model. The burn is induced by applying a heated metal element to the back of the *G. mellonella*. Shortly after the burn wound can be topically infected with a chosen micro-organism. Any treatments can be applied shortly after the procedure. The larvae are then incubated and monitored to observe the survival rates. (Adapted from Maslova *et al*., 2021 [21].)

Compared to more conventional wounds such as lacerations, the nature of burn wounds is complex due to the associated multi-system damage; as a result, they are impossible to accurately recreate and study *in vitro* [16]. Multiple *in vivo* models have been established to study the burn wound and infection, one of the most widely used models being the murine model. The other *in vivo* models include porcine, canine and rabbit ear model [17–19]. These models provide invaluable insights into the burn physiology and pathology and are essential for the generation of robust pre-clinical data. These models have several advantages to them, ranging from body system similarities to the versatility of the model organism. However, they have a few disadvantages in common such as (1) they are associated with a high level of ethical and animal welfare concerns; (2) the size of the experimental cohort is very limited; (3) they are costly; (4) they are not suitable for screening-based approaches. In addition to that, the burn wound injury is a severe and morbid condition, which is very distressing to the animal [20]. This creates additional hurdles for researchers and therefore limits *in vivo* burn wound research. Recently, an invertebrate burn wound, and infection model has been established using *Galleria mellonella* [21] (Fig. 1). *G. mellonella* is a robust animal model that has gained significant popularity in the last decade among researchers. It has been firmly established for use in drug toxicity and virulence assays for a wide range of pathogens [22–24]. The *G. mellonella* burn wound model follows important hallmarks of burn wound trauma and infection, for example, the decrease in survival with the increase of the burn surface area and a significant decrease in survival after topical burn wound infection. Using this invertebrate in burn research addresses several hurdles presented by larger mammalian models such as affordability, cohort sizes, and the ethical and welfare concerns and the need for ethical approval [25]. It also enables high-throughput screening, which is not possible with mammalian burn wound models.

This study validates *G. mellonella* as a model for studying probiotic treatments in a burn wound infection, as well as demonstrating that this model can facilitate inoculation with multiple micro-organisms. This will help fast track the development of novel probiotic-based wound solutions by enabling high-throughput screening of potential candidate probiotics as well as mechanism of action studies. The use of this model in probiotic wound therapeutic development will also help to refine and reduce the number of mammals used in downstream studies.

## Methods

### Bacterial strains and inoculum preparation

Strains of *Lactobacillus* spp. *L. reuteri* CCUG44144*, L. casei* CCUG2145T*, L. jensenii* CCUG35572*, L. reuteri* CCUG33624*, L. fermentum* CCUG30138*, Lactobacillus crispatus* CCUG42898 and *Lactobacillus gasseri* CCUG44046 purchased from Culture Collection University of Gothenburg) and *P. aeruginosa* PA14 were stored as 20 % glycerol stocks at −80 °C until required [21]. *P. aeruginosa* was inoculated into a universal 30 ml tube with 5 ml of lysogeny broth and incubated overnight at 37 °C at 180 r.p.m. until it reached OD_600_=~3.0. *Lactobacillus* strains were inoculated into 50 ml centrifuge tubes and Petri dishes with De Man, Rogosa and Sharpe (MRS) nutrient media. The cultures were placed into a hermetic chamber with an anaerobe gas generation sachet and incubated anaerobically for 48–72 h at 37 °C until the liquid cultures reached OD_600_=~1.5.

### Preparation of cell-free *Lactobacillus* spp. supernatant

*Lactobacillus* spp. 48–72 h liquid cultures were centrifuged for 10 mins at 4500 g at room temperature. The obtained supernatant was filter-sterilized with 0.2 nm filter and the bacterial pellet was discarded. The prepared supernatant was used the same day.

### Animal acquisition and preparation

*G. mellonella* were obtained from a pet-food supplier (LiveFood UK, Somerset, United Kingdom) in plastic containers with wood shavings where they were kept before the experiments. Prior to use, the larvae were stored at +4 °C to minimize the larval movement during procedure. The larvae were sorted into Petri dishes lined with filter paper ensuring all larvae are above 200 mg in weight, which is consistent with them reaching full adulthood and show no signs of melanization (black markings). Only 10 larvae per dish were permitted.

### *In vivo* burn wound induction and *P. aeruginosa* infection establishment

Overall, 70 % ethanol was used to sterilize the larval body surface spraying the entire larval body with the solution (Fig. 2.1, Video S1, available in the online version of this article). The Petri dishes were left open in a sterile environment to allow for the ethanol to evaporate after sterilization. A *G. mellonella* larva was placed on its ventral side to allow access to the back segment and held down by its head and thorax segments. The burn instrument (a steel nail with a head size of 2 mm^2^ embedded in cork) was heated in the middle flame of the Bunsen burner until red/white-hot and applied to the middle segment of *G. mellonella* back for 4 s (Fig. 2.2, Video S2). Any larvae that showed major haemolymph loss or protruding fat body after the procedure was immediately euthanized by placing it at −20 °C for at least 20 min to minimize the suffering. Immediately after the burn is established, 10 µl of overnight *P. aeruginosa* PA14 culture was pipetted on top of the wound (Fig. 2.5, Video S3). The larvae were allowed to rest for 10 min before introducing any further treatments.

**Fig. 2.**
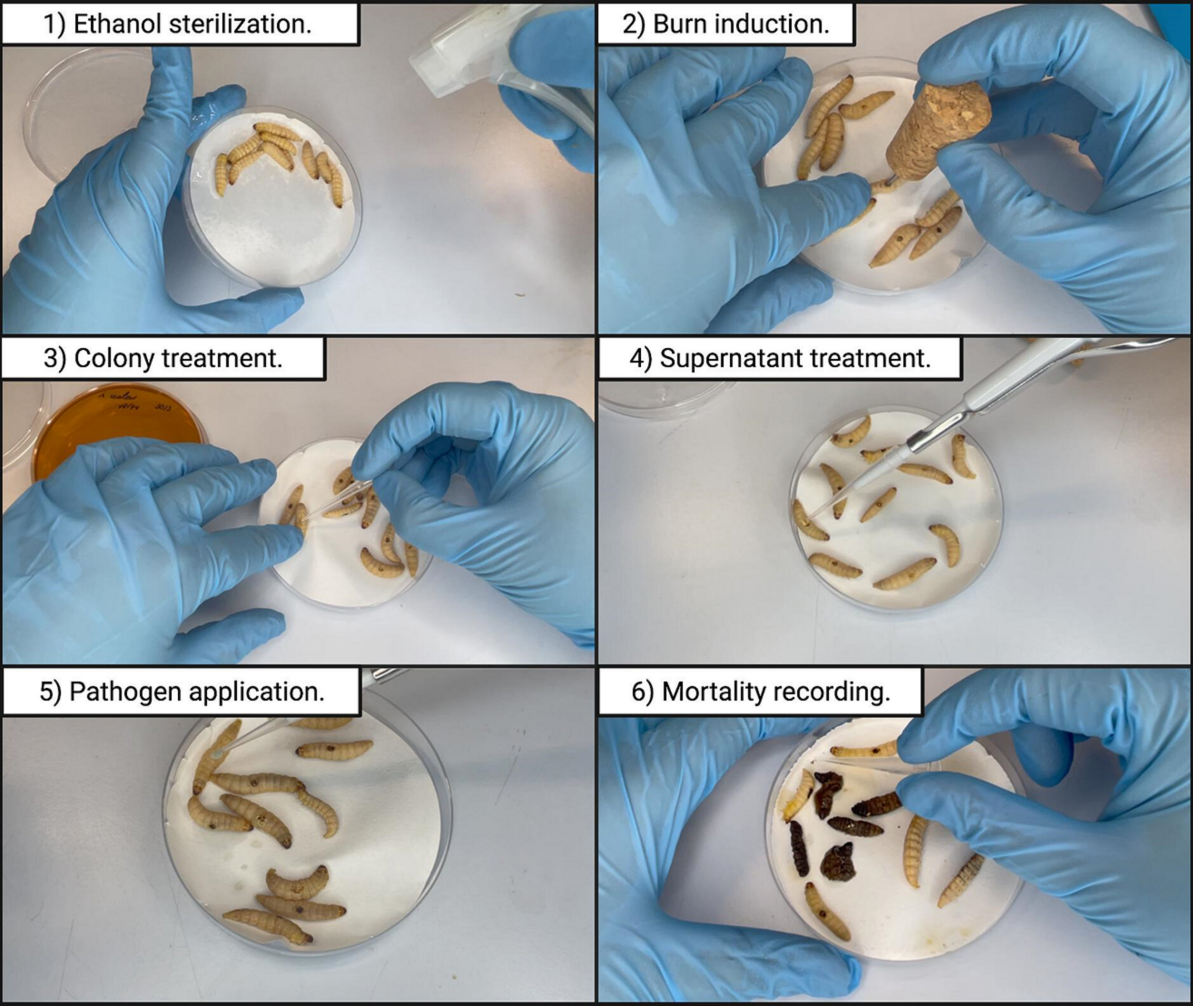
A step-by-step outline of the protocol used for the *G. mellonella* burn wound and infection assay. (1) The larval body is sterilized with 70 % ethanol. (2) The burn is induced by the application of a heated metal element to the back of the larvae. (3), (4) A colony or cell-free supernatant is applied to the established burn wound. (5) The wound is topically inoculated with the pathogen. (6) The mortality is recorded by gently agitating the larvae with a pipette tip to elicit a motility response. Full videos of each step are available as Supplementary Material, available with the onine version of this article.

### *In vivo L. reuteri* CCUG44144 colony and *Lactobacillus* spp. supernatant treatments of *P. aeruginosa* PA14 infected wound

After inducing the burn, a sterile 200 µl pipette tip was used to transfer a colony of *L. reuteri* CCUG44144 from the MRS agar plate to the wound. The tip was gently brushed against the wound to minimize mechanical damage to the wound (Fig. 2.3, Video S4). Following that, 10 µl of an overnight culture of *P. aeruginosa* PA14 was applied onto the treated wound (Fig. 2.5). In the supernatant treatment experiments, 10 µl of cell-free *Lactobacillus* spp. supernatant was applied onto the burn wound immediately prior to the establishment of *P. aeruginosa* PA14 infection (Video S5), as well as to the groups that were not infected with *P. aeruginosa* (Fig. 2.4). The control groups received no treatment post-burn induction. The larvae were incubated at 37 °C for 72 h. The mortality was recorded every hour. Mortality was recorded upon complete loss of larval movement even with external stimulation (Fig. 2.6, Video S6). Supernatant and colony treatment experiments were performed on different dates with different treatment groups.

### *In vivo* tetracycline treatment of the burn wound infected with *P. aeruginosa* PA14

A 2.2 mg ml^−1^ tetracycline solution, a concentration that is in range with therapeutic topical tetracycline ointment concentrations, was prepared [26–28]. After the establishment of burn wound and infection with *P. aeruginosa* PA14 in *G. mellonella*, 10 µl of 2.2 mg ml^−1^ tetracycline solution was applied on top of the wound. The larvae were incubated at 37 °C for 72 h.

## Results

### *L. reuteri* colonization and supernatant treatment improve *G. mellonella* survival

**Fig. 3.**
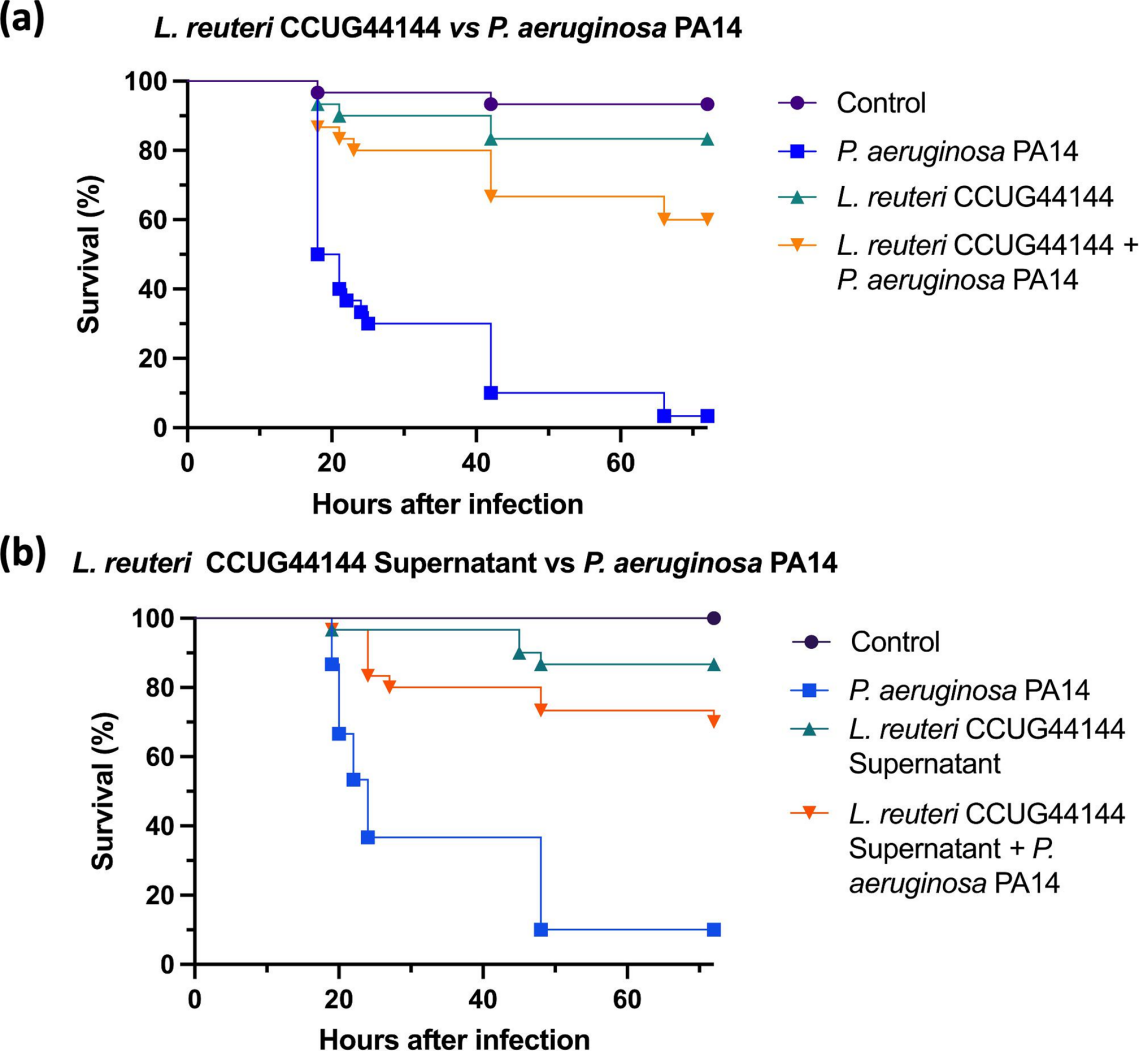
Survival curves of *in vivo* burn wound treated with *L. reuteri* CCUG44144 colonies (**a**) and supernatant (**b**) in the topical infection with *P. aeruginosa* PA14, *n*=30. (**a**) *P. aeruginosa* PA14 *vs L. reuteri* CCUG44144 *+ P. aeruginosa* PA14 survival *Log rank P value<0.0001*. (**b**) *P. aeruginosa* PA14 *vs L. reuteri* CCUG44144 *Supernatant + P. aeruginosa* PA14 survival *Log rank P value<0.0001*. Statistical significance was determined with Log rank statistical test with Bonferroni-corrected threshold.

Initially, the *G. mellonella* larvae burn wound was seeded with *L. reuteri *, which has been reported to have an antimicrobial activity against *P. aeruginosa* via the production of reuterin [29, 30]. *L. reuteri* has also been shown to augment the healing processes in rats [31]. After the inoculation of the wound with *L. reuteri* colonies, the wound was subsequently infected with *P. aeruginosa *. Negative controls and *L. reuteri* CCUG44144 only inoculated groups exhibited 10 % or less mortality rates, which aligns with this strain being a commensal micro-organism. *P. aeruginosa* infected larvae exhibited above 90 % mortality, which correlates to the expected mortality rates of this clinical isolate [21]. The experimental group with wounds inoculated with *L. reuteri* CCUG44144 and infected with *P. aeruginosa* exhibited a significant reduction in mortality of 55 % in comparison to *P. aeruginosa* only infections (Fig. 3a).

Following the abovementioned findings, *L. reuteri* supernatant was tested against *P. aeruginosa* infection to determine if secreted factors were responsible for the observed reduction in virulence. *G. mellonella* larvae burn wounds were infected with *P. aeruginosa* and *L. reuteri* supernatant was topically applied. Negative controls exhibited less than 10 % mortality. Positive controls of *P. aeruginosa* PA14 infection exhibited more than 90 % mortality. Control groups treated with just *L. reuteri* supernatant exhibited less than 20 % mortality (Fig. 3). Groups treated with *L. reuteri* supernatant prior to *P *. *aeruginosa* infection showed a significant 60 % reduction of mortality after the treatment (Fig. 3b). This aligned with the findings observed from the colony treatment and the previously reported effects of *L. reuteri* on *P. aeruginosa* pathogenicity [29, 30].

### *Lactobacillus* spp. supernatant improves the survival of *G. mellonella* after *P. aeruginosa* infection

**Fig. 4.**
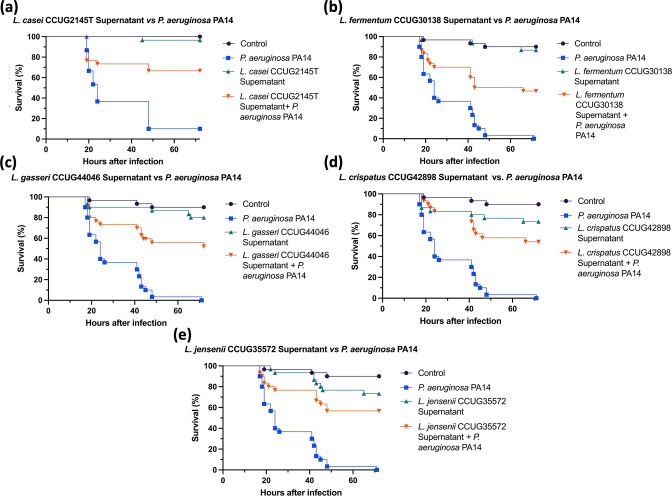
Survival curves of *in vivo* burn wound *P. aeruginosa* PA14 infection treated with *Lactobacillus spp* supernatants, *n*=30. (**a**) *P. aeruginosa* PA14 versus *L. casei* CCUG2145T, Log rank *P* value<0.0001. (**b**) *P. aeruginosa* PA14 versus *L. fermentum* CCUG30138, Log rank *P* value<0.0001. (**c**) *P. aeruginosa* PA14 versus *L. gasseri* CCUG44046, Log rank *P* value<0.0001. (**d**) *P. aeruginosa* PA14 versus *L. crispatus* CCUG42898, Log rank *P* value<0.0001. (**e**) *P. aeruginosa* PA14 versus *L. jensenii* CCUG35572, Log rank *P* value<0.0001. *L. casei* treatment experiment was performed on the same day as *L. reuteri* treatment in Fig. 3(b), resulting in the difference in the ‘Control’ and ‘* P. aeruginosa* PA14’ control curves between supernatant treatments. Statistical significance was determined with Log rank statistical test with Bonferroni-corrected threshold.

Due to the probiotic effects observed in the larvae treated with *L. reuteri* colonies and supernatant, more *Lactobacillus* strains supernatants were tested against *P. aeruginosa* burn wound infection in *G. mellonella*. The selected strains have been previously reported to have antimicrobial or probiotic effects in burn wound infections. Groups with *P. aeruginosa* infected burns showed a significant 60 % reduction of mortality after the treatment with *L. casei* (Fig. 4a). *L. casei* has been previously reported to interfere with the adhesion mechanisms of *P. aeruginosa* in Wistar rats [32]. Infected burn group treated with *L. gasseri* showed a significant 55 % reduction in mortality, which aligns with reported findings in murine burn infection [33]. *P. aeruginosa* infected burn wounds treated with *L. crispatus* and *L. jensenii* exhibited a significant 50 % reduction in mortality (Fig. 4b, e). However, *L. crispatus* supernatant only treated group exhibited a 30 % mortality, which is higher than the expected findings (Fig. 4b). *L. fermentum* treatment resulted in a significant 40 % reduction of mortality (Fig. 4d). Overall, all of the selected strains exhibited a significant reduction of mortality in *P. aeruginosa* burn wound infection, which is in line with the reported findings in *ex vivo* and *in vivo* models [13–15, 32]. An important observation however is that this model was able to distinguish differences in the probiotic potential of the different strains highlighting its versatility.

### *Lactobacillus* colonization matches antibiotic activity of tetracycline

**Fig. 5.**
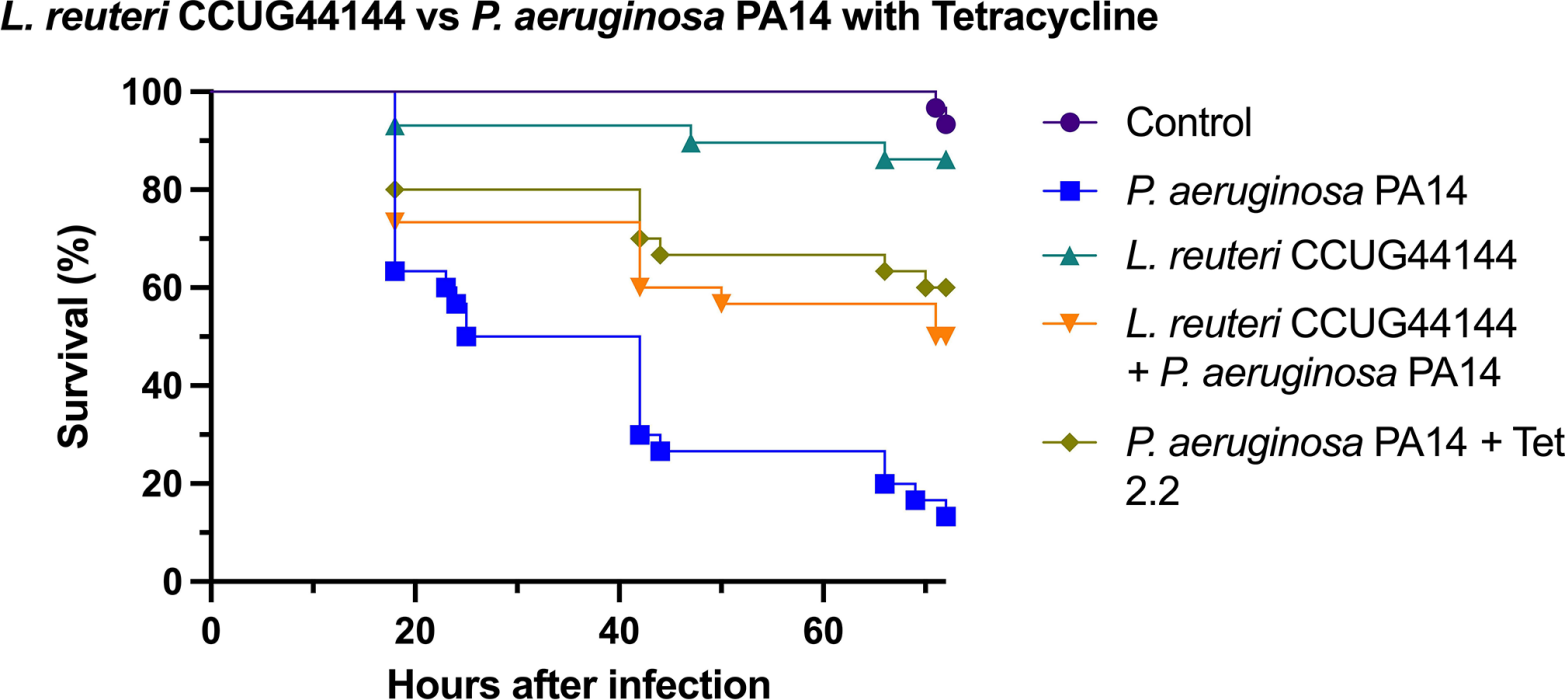
Survival curves of *in vivo* burn wound *P. aeruginosa* PA14 infection treated with *L. reuteri* CCUG44144 and topical application of 2.2 mg ml^−1^ solution of tetracycline, *n*=30. *P. aeruginosa* PA14 *vs L. reuteri* CCUG44144 *+ P. aeruginosa* PA14 survival *Log rank P value<0.01. P. aeruginosa* PA14 *vs P. aeruginosa* PA14 *+* Tet 2.2 mg ml^−1^ survival *Log rank P value<0.0001. P. aeruginosa* PA14 *+* Tet 2.2 mg ml^−1^ *vs L. reuteri* CCUG44144 *+ P. aeruginosa* PA14 survival *Log rank P value>0.01*. Statistical significance was determined with Log rank statistical test with Bonferroni-corrected threshold.

To challenge the model further and compare the probiotics effects to that of a topical antibiotic treatment, *L. reuteri* CCUG44144 was tested against *P. aeruginosa* with topical tetracycline treatment (Fig. 5). Topical tetracycline treatment is one of the common clinical strategies against wound infection [34]. The addition of topical tetracycline treatment improved the survival of larvae infected with *P. aeruginosa* by almost 50 %, which suggests in this model at least, that probiotic prophylaxis is as effective as antibiotic therapy. There was no significant difference observed between tetracycline treated larvae and *L. reuteri* treated larvae survival, which validates the effectiveness of *L. reuteri* treatment in *G. mellonella* burn wound model against *P. aeruginosa *.

## Discussion

This invertebrate burn wound and infection model using *G. mellonella* addresses several issues that affect *in vivo* burn wound research, such as limited cohort sizes, strict ethical considerations, affordability and handling and maintenance. The protocol described in this study validates this model as a tool to determine the *in vivo* efficacy of burn wound probiotic treatments as well as its capacity to be used to study interspecies bacterial interactions. The results obtained from this protocol, demonstrating the probiotic potential of *Lactobacillus* spp. and their supernatants in the burn wound microenvironment, align with previously reported findings [14, 35]. Despite the previously described advantages this model has several limitations that need to be considered carefully. The anatomy of larval cuticle consists of multiple layers, but indisputably is very different from mammalian skin structure [36]. Even though, its innate immune system has similar elements to its mammalian counterpart, *G. mellonella* also lacks an adaptive immune system, which plays a major role in the burn trauma and infection pathogenesis [37]. However, due to the challenges associated with currently established *in vivo* burn wound and infection models, *G. mellonella* burn wound and infection model could reduce and refine the use of the larger mammalian models as preliminary experiments to optimize the dosing, formulations and timings can be performed in the invertebrate model. *G. mellonella* burn wound model has been used to assess the effects of antibiotic wound treatments and other antimicrobial therapies against burn wound infections [38–40]. This model will also facilitate high-throughput screening of the commensal skin microbiota to identify potential probiotic strains *in vivo*, something that is not possible with traditional mammalian models.

*Lactobacillus* spp. have long been established as one of the most prolific probiotic bacteria. Several mechanisms have been associated with their therapeutic effects, such as their impact on local pH and the production of antimicrobial compounds such as reuterin [29, 41]. Several species of *Lactobacillus* have been reported to produce bacteriocins, which exhibited an antimicrobial effect on multidrug-resistant *P. aeruginosa* wound isolates [42]. *L. acidophilus* and *L. casei* can produce surfactants that can reduce *S. aureus* and *Staphylococcus epidermidis* biofilm development and induce dispersal [43, 44]. The therapeutic effects of *Lactobacillus* treatments observed in this study could be attributed to several antimicrobial mechanisms deployed by *Lactobacillus* spp. In the *L. reuteri* colony treatment (Fig. 3a) some of its therapeutic effect can also be attributed to competitive exclusion within the wound bed as *L. reuteri* has been previously shown to inhibit *S. aureus* infection via the same mechanism [14]. Overall, all of the tested *Lactobacillus* strains have been previously reported for producing antimicrobial compounds or exhibiting antimicrobial or anti-virulence activity against major wound pathogens via multiple mechanisms [15, 32, 33, 45, 46]. This aligns with the therapeutic effects seen when testing the supernatants in Figs 3(b) and 4.

There are several crucial aspects that must be attended to when using this protocol. Larval sizes and health at the beginning of the experiment are important to the survival rates downstream [47]. Larvae that exhibit any sign of melanization or appear to be flaccid upon retrieval from +4 °C incubation should not be used as it is an indication of their declining health condition and will affect their survival [48]. During the burn procedure, attention must be paid to how much haemolymph is lost in the process. Haemolymph should appear as transparent pale-yellow fluid. Due to *G. mellonella*’s open circulatory system major haemolymph loss will result in an early death from the dehydration [49]. Additionally, due to the placement of the burn wound on the dorsal side of larvae it comes in close proximity with the internal systems of the invertebrate. Therefore, any sign of protruding tissues or leakage of non-transparent fluid from the wound should lead to immediate euthanasation of the animal via incubation at −20 °C. During the step of colony application to the wound, special attention needs to be paid upon touching the wound with the pipette tip. Larval cuticle is thin and fragile, which is only amplified after the burn trauma. In addition to that, the source of *G. mellonella* larvae needs to be considered. The experiments conducted in this study were performed on shop-grade larvae, meaning that the diet and rearing conditions of the larvae could not be controlled. Using in-house reared or research-grade larvae could limit these variables.

During the application of liquid treatments or inoculating the wound with liquid pathogen cultures, the applications need to be spaced out. *G. mellonella* forms an eschar on top of the burn wound, which provides a physical protection to the wound. Applying multiple liquid elements to this area in a short period of time could lead to increased permeability of the scab and cause early decline of the larval condition. In addition, during the sterilization step, using any other sterilization agent such as industrial methylated spirits should be avoided and a sufficient time should be allocated for the ethanol to evaporate from the filter paper in the Petri dish to avoid larval alcohol poisoning.

## Supplementary Data

Supplementary material 1

Supplementary material 2

Supplementary material 3

Supplementary material 4

Supplementary material 5

Supplementary material 6
